# The costs of nosocomial resistant gram negative intensive care unit infections among patients with the systemic inflammatory response syndrome- a propensity matched case control study

**DOI:** 10.1186/s13756-015-0045-8

**Published:** 2015-02-02

**Authors:** Anupama Vasudevan, Babar Irfan Memon, Amartya Mukhopadhyay, Jialiang Li, Paul Ananth Tambyah

**Affiliations:** Yong Loo Lin School of Medicine, National University of Singapore, 10 Medical Drive, Singapore, 117597 Singapore; Steward Carney Hospital, Tufts University School of Medicine, 136 Harrison Avenue, Boston, MA 02110 USA; Division of Respiratory and Critical Care Medicine, National University Health System, 1E Kent Ridge Road, Singapore, 119228 Singapore; Department of Statistics and Applied Probability, National University of Singapore, Faculty of Science, 6 Science Drive 2, Singapore, 119077 Singapore; Division of Infectious Diseases, National University Health System, 1E Kent Ridge Road, Singapore, 119228 Singapore

**Keywords:** Resistant gram negative infection, Sensitive gram negative infection, Critically ill patient, ICU, Costs

## Abstract

**Background:**

Infections due to multi-drug resistant gram negative bacilli (RGNB) in critically ill patients have been reported to be associated with increased morbidity and costs and only a few studies have been done in Asia. We examined the financial impact of nosocomial RGNB infections among critically ill patients in Singapore.

**Methods:**

A nested case control study was done for patients at medical and surgical ICUs of a tertiary university hospital (August 2007-December 2011) matched by propensity scores. Two groups of propensity-matched controls were selected for each case patient with nosocomial drug resistant gram negative infection: at-risk patients with no gram negative infection or colonization (Control A) and patients with ICU acquired susceptible gram negative infection (SGNB) (Control B). The costs of the hospital stay, laboratory tests and antibiotics prescribed as well as length of stay were compared using the Wilcoxon matched-pairs signed rank test.

**Results:**

Of the 1539 patients included in the analysis, 76 and 65 patients had ICU acquired RGNB and SGNB infection respectively. The median(range) total hospital bill per day for patients with RGNB infection was 1.5 times higher than at-risk patients without GNB infection [Singapore dollars 2637.8 (458.7-20610.3) vs. 1757.4 (179.9-6107.4), p0.0001]. The same trend was observed when compared with SGNB infected patients. The median costs per day of antibiotics and laboratory investigations were also found to be significantly higher for patients with RGNB infection. The length of stay post infection was not found to be different between those infected with RGNB and SGNB.

**Conclusion:**

The economic burden of RGNB infections to the patients and the hospital is considerable. Efforts need to be taken to prevent their occurrence by cost effective infection control practices.

## Background

Critically ill patients have been shown to have higher risk of nosocomial infection compared to other hospitalized patients [1]. Intensive Care units have been identified as the epicenter of not just nosocomial infections but also of drug resistant infections [2]. These difficult to treat drug resistant organisms were named as “ESKAPE” pathogens [3] or in the words of the Infectious Diseases Society of America as “Bad bugs, no drugs”. While resistance among gram positive isolates has been stable or decreasing globally, it has been rising among nosocomial gram negative isolates [4]. This is especially so in the ICU [5]. Organisms such as Klebsiella spp*.*, *Escherichia Coli, Acinetobacter baumanii*, *Pseudomonas aeruginosa* and *Stenotrophomonas maltophilia* often have decreased susceptibility to many antibiotics making them difficult to treat [6]. In Singapore, third generation cephalosporin resistant *Klebsiella pneumoniae* were found to be the predominant organisms among the intensive care unit (ICU) isolates in a recent study [7].

Nosocomial infections in general are associated with increased hospital costs, causing a financial burden to the patient and society [8]. With high rates of drug resistance they are believed to have a significant economic impact [9]. Even among less severely ill patients, drug resistant infections increase total hospitalization costs [10]. Studies which documented costs of nosocomial resistant gram negative bacilli (RGNB) infections have generally considered site or organism specific RGNB infections and often included all hospitalized patients [11,12]. To our knowledge, very little has been published addressing the costs of nosocomial RGNB infections in ICUs.

Most of the previous studies are case control or observational studies where obtaining a comparable group of controls is critical. Matching by use of specific variables may not completely help eradicate bias as there may be other confounding factors not matched for and it is also possible to “over-match” cases and controls. It has been shown that matching by propensity scores results in better selection of a comparator group with less bias [13,14]. Propensity scores aid in estimating the likelihood of patients in the ICU being infected with RGNB based on a risk factor analysis thus taking multiple relevant risk factors into consideration.

We therefore designed the current study to estimate the excess cost associated with nosocomial ICU acquired RGNB infection (any site) among critically ill patients using a propensity score matched case–control approach.

## Methods

### Setting

A nested case control study was performed within a prospective cohort study conducted at the medical and surgical ICU of a 1000-bed tertiary hospital affiliated to the National University of Singapore from August 2007 through December 2011 [15]. The prospective study included all consecutive adult patients over 21 years of age who had been admitted for more than 24 hours to either the medical or surgical ICU. The following data were collected prospectively for each patient: demographics, co-morbidities, APACHE II, Charlson scores, invasive devices, antibiotics used, any surgical procedure performed, culture results and total hospitalization costs along with individual costs of laboratory and radiological investigations, medications including antibiotics.

The study was approved by the Institutional Domain Specific Review Board (B/06/140) with a waiver of consent.

### Definition

Resistant Gram Negative Bacilli (RGNB): *Acinetobacter baumannii*, *Pseudomonas aeruginosa*, *Klebsiella pneumoniae*, *Escherichia coli* were the organisms of interest: Multi-drug resistance in these organisms was defined as being non susceptible to > =1 agent in > =3 antimicrobial categories based on the European Centers of Disease control categorization [16].

Colonization: All patients with RGNB cultured from any clinical specimen with no associated clinical signs or symptoms of infection with no treatment initiated by the clinician, or documentation as colonization by an infectious disease specialist [17].

Infection: All patients with RGNB cultured from any of the clinical specimens who were treated for RGNB infection according to the National Health and Safety Network’s definitions of infection [17].

Nosocomial ICU acquired RGNB infection: RGNB isolated after 48 hours of admission to the ICU.

Costs: Total hospitalization costs were obtained from the hospital financial records based on charges paid by the patient or their insurers inclusive of government subsidies.

Cases: Patients with Nosocomial ICU acquired RGNB infection

Controls: Patients with no GNB infection or colonization (Control A) or Nosocomial ICU acquired SGNB infection (Control B).

### Selection of cases and controls - propensity score matching

Propensity score calculation: Propensity scores were calculated using the independent risk factors for Nosocomial RGNB infection acquired in the ICU identified in our study by a multivariable logistic regression analysis. Gender was also included in the final model for calculation of propensity scores.

### Selection of controls

Two sets of controls were selected for these case patients.

At-risk patients with no GNB infection or colonization (Control A): patients with nosocomial ICU acquired RGNB infection (case) were matched with patients with no GNB infection or colonization (control A) by the nearest neighbor matching method without replacement in a ratio of 1:1.

Patients with nosocomial ICU acquired SGNB infection (Control B): patients with nosocomial ICU acquired RGNB infection with no previous SGNB infection/colonization this admission (case) were matched with patients with nosocomial ICU acquired SGNB infection (control B) by the nearest neighbor matching method with replacement in a ratio of 1:1. We employed matching with replacement as the numbers of patients with SGNB infections were limited. *Controls (control B) were matched for cases with no previous SGNB infection/colonization this admission in order to control for the compounding treatment costs of a previous SGNB infection.*

Balance checks were conducted between the propensity score matched cases and controls to ensure quality matching so as to decrease the bias.

### Calculation of costs

The costs per day were used in order to correct for the effects of mortality which may shorten the length of stay of the most severely ill patients.

Total hospital costs per day: For each case and each control, the total bill for the entire hospital stay was obtained through the hospital finance system and was divided by the number of total hospital stay in days to obtain the hospital costs per day.

Costs per day from date of ICU admission: We obtained the costs from date of ICU admission for each patient and divided them by the number of hospital days counting from ICU admission.

Costs per day from date of infection: For each case and Control B (SGNB infection), the costs were obtained from date of infection and were divided by the number of days the patient stayed in the hospital from the date of infection.

The same approach was repeated for obtaining the costs per day for the different categories.

### Length of stay analysis

Including the ICU survivors of the nosocomial RGNB infection, we investigated differences in the length of stay between the cases and the 2 different controls. Hospital stay from the date of admission to ICU was used in the analysis for those without infection. In addition, we also compared the length of hospital stay post infection for cases and Control B (SGNB infection).

### Statistical analysis

Analysis was done using STATA 10.1(STATA Corp, Texas, USA). The costs between the cases and controls (A & B) were compared using student’s paired t-test or Wilcoxon matched-pair signed rank tests where applicable. Separate analyses were done to compare cases with the two different control groups.

## Results

**Figure 1:**
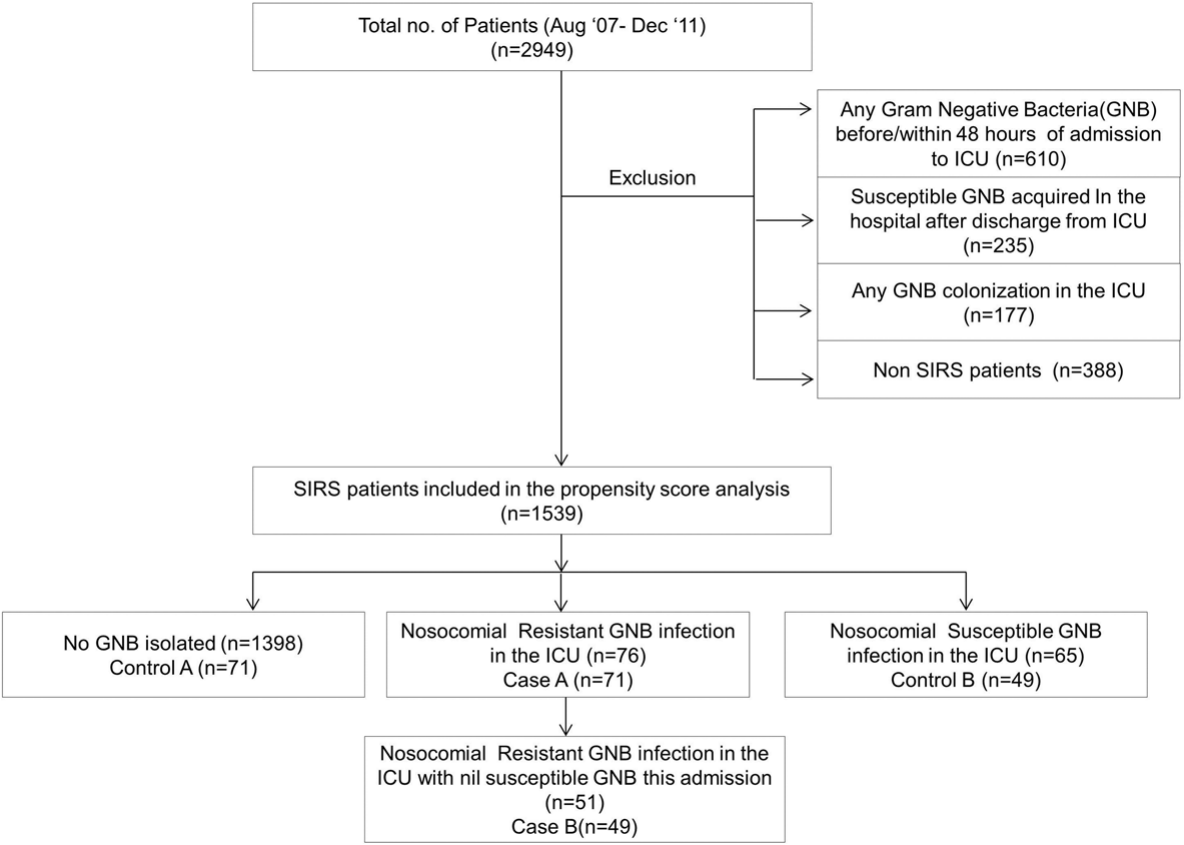
**Patient inclusion chart.**

A total of 2949 patients were enrolled in our study. Excluding patients with a GNB isolate before or within 48 hours of admission to the ICU, any GNB colonization in the ICU and those with SGNB isolates after discharge from the ICU and non-SIRS patients, a total of 1539 patients were included in the analysis. Of these, 76 and 65 patients had a laboratory confirmed nosocomial ICU acquired RGNB and SGNB infection respectively (Figure 1).

### Propensity score calculation

Among patients with SIRS, we identified the following risk factors to be independently associated with the occurrence of nosocomial ICU acquired RGNB infection when compared to patients with no GNB colonization or infection by logistic regression analysis- a stay of more than 5 days in the ICU, presence of any GNB, administration of carbapenems in the past 6 months, having a surgical procedure during this admission and end stage renal disease on dialysis [18]. Gender was added to this model and propensity scores were then calculated [14].

Seventy-one out of 76 case patients had complete details of the costs for their admission and were analyzed.

### Control selection

Control A: 71 control patients were chosen from the cohort of patients with SIRS and no GNB infection/colonization (n = 1398) using the propensity scores obtained from the logistic model. One control per case was chosen without replacement.

Control B: Of the 76 case patients with RGNB infection, 51 of them had no SGNB isolated this admission prior to the RGNB infection and were further analyzed for this case control portion of the study. Controls were chosen from the cohort of patients with ICU acquired SGNB infection (n = 65) in a ratio of 1:1 with replacement. 49 of the 51 cases had complete cost details. Table 1 and Table 2 shows the characteristics of cases and controls involved in the two analyses.

**Table 1:** **Patient characteristics: analysis A: cases and propensity matched uninfected controls (controls A)**

	**Cases (n = 71)**	**Controls (n = 71)**	**p value**
Gender			0.49
Male	43 (60.6%)	47 (66.2%)	
Female	28 (39.4%)	24 (33.8%)	
Intensive care unit (ICU)			0.11
Medical ICU	45 (63.4%)	35 (50%)	
Surgical ICU	26 (36.6%)	35 (70%)	
Admission to the ICU			0.61
Direct admission	35 (49.3%)	38 (53.5%)	
Transfer from the ward	36 (50.7%)	33 (46.5%)	
Diabetes	27 (38%)	21 (29.6%)	0.29
Renal impairment	17 (23.9%)	13 (18.3%)	0.41
Cerebrovascular accident	7 (9.9%)	12 (16.9%)	0.22
Peptic ulcer disease	4 (5.6%)	0 (0%)	0.12
Myocardial infarction	12 (16.9%)	10 (14.1%)	0.64
Congestive cardiac failure	1 (1.4%)	4 (5.6%)	0.37
Liver disease	2 (2.8%)	3 (4.2%)	1
Malignancy	14 (19.7%)	8 (11.3%)	0.61
Central venous catheter	43 (60.6%)	54 (76.1%)	0.08
Endotracheal intubation	66 (92.9%)	71 (100%)	0.09
Mean days of duration of mechanical ventilation (SD)	5.8 (SD4.7)	4.7 (SD3.9)	0.14
Prior SGNB** infection or colonization in the previous 6 months months	18 (25.4%)	21 (29.6%)	0.57
Prior Carbapenems in the past 6 months	42 (59.2%)	38 (53.5%)	0.61
Prior 3rd generation Cephalosporins in the past 6 months	46 (64.8%)	36 (50.7%)	0.12
Surgery prior to RGNB*** infection	35 (49.3%)	39 (54.9%)	0.61
Stay in ICU for more than 5 days	51 (71.8%)	55 (77.5%)	0.56
Mortality	36 (50.7%)	18 (25.4%)	0.003
Mean age (SD)	60.6 (SD1.8)	59.1 (SD17.9)	0.58
Mean APACHEII (SD)	18.1 (SD7.3)	19.6 (SD7.7)	0.24
Mean age adjusted Charlson score (SD)	3.4 (SD2.9)	3.1 (SD2.9)	0.56
Median PreICU stay (range)	1 (0–40)	0 (0–29)	0.54
Mean propensity scores (SD)	0.15 (SD0.2)	0.14 (SD0.2)	0.92

**Sensitive Gram Negative Bacilli ***Resistant Gram Negative Bacilli.

**Table 2:** **Patient characteristics: analysis B: cases and propensity matched controls infected with SGNB (controls B)**

	**Cases (n = 49)**	**Controls B (n = 49)**	**p value**
Gender			0.83
Male	32 (65.3%)	31 (63.3%)	
Female	17 (34.7%)	18 (36.7%)	
Intensive care unit (ICU)			0.61
Medical ICU	26 (53.1%)	22 (47.8%)	
Surgical ICU	23 (46.9%)	24 (52.2%)	
Admission to the ICU			0.54
Direct admission	27 (55.1%)	24 (48.9%)	
Transfer from the ward	22 (44.9%)	25 (51.1%)	
Diabetes	15 (30.6%)	13 (26.5%)	0.66
Renal impairment	9 (18.4%)	13 (26.5%)	0.33
Cerebrovascular accident	5 (10.2%)	12 (24.5%)	0.11
Peptic ulcer disease	3 (6.1%)	1 (2.1%)	0.62
Myocardial infarction	8 (16.3%)	9 (18.4%)	0.79
Congestive cardiac failure	3 (6.1%)	0 (0%)	0.24
Liver disease	2 (4.1%)	1 (2.1%)	1
Malignancy	4 (8.2%)	0 (0%)	0.12
Central venous catheter	45 (91.8%)	45 (91.8%)	1
Endotracheal intubation	49 (100%)	47 (95.9%)	0.49
Mean days of duration of mechanical ventilation (SD)	7.9 (SD6.8)	7.6 (SD2.9)	0.80
Prior SGNB** infection or colonization in the past 6 months	3 (6.1%)	0 (0%)	0.24
Prior Carbapenems in the past 6 months	23 (46.9%)	23 (46.9%)	1
Prior 3rd generation Cephalosporins in the past 6 months	25 (51.1%)	15 (30.6%)	0.05
Surgery prior to RGNB*** infection	23 (46.9%)	27 (55.1%)	0.42
Stay in ICU for more than 5 days	38 (77.6%)	37 (75.5%)	0.81
Mortality	28 (57.1%)	15 (30.6%)	0.008
Mean age (SD)	59.5 (SD18.2)	57.1 (SD16.4)	0.5
Mean APACHEII (SD)	20.4 (SD7.2)	20.6 (SD5.8)	0.88
Mean age adjusted Charlson score (SD)	3.5 (SD3.1)	3.2 (SD2.7)	0.62
Median PreICU stay (range)	0 (0–25)	1 (0–32)	0.21
Mean propensity scores (SD)	0.58 (SD0.24)	0.58 (SD0.24)	0.99

**Sensitive Gram Negative Bacilli ***Resistant Gram Negative Bacilli.

### Cost analysis

A Wilcoxon matched pair signed rank test was done to compare the costs per day for the propensity matched cases and controls for both the analyses.

Comparison of the costs between patients with RGNB infection and no GNB infection (Table 3) showed that the median total hospital costs per day and the costs in all the categories especially the cost of carbapenems were significantly higher for cases.

**Table 3:** **Analysis A: comparison of costs between the propensity matched cases and controls A**

**Costs per day (S$)**	**RGNB (n = 71)**	**no GNB (n = 71)**	**p value**
Median total hospitalization costs (range)	2637.8 (458.7-20610.3)	1757.4 (179.9-6107.4)	0.0001
**After admission to the ICU**			
*Median laboratory costs (range)*	381.7 (68.9-3366.5)	252.2 (0.06-1037.8)	<0.001
*Median investigation costs (range)*	145.3 (27.8-1663.8)	108.7 (0–452.6)	0.007
*Median total medication costs (range)*	332.7 (48.3-8625.1)	147.6 (0.08-1478.9)	<0.001
*Median antibiotics costs (range)*	69.9 (0.93- 334.7)	32.3 (0–305.4)	0.001
*Median carbapenem costs (range)*	22.31 (0–223.53)	0 (0–210.2)	<0.001

Similarly, comparing cases with RGNB infections with their propensity matched control patients with SGNB infection (Control B), we found that the median costs per day of for RGNB infected patient were significantly higher in all the categories (Table 4).

**Table 4:** **analysis B: comparison of costs between the propensity matched cases and controls B**

**Costs per day (S$)**	**RGNB (n = 49)**	**SGNB (n = 49)**	**p value**
Median total hospitalization costs (range)	2795.9 (506.9-4882.3)	2009.6 (857.5-5213.5)	0.0004
**After admission to the ICU**			
*Median laboratory costs (range)*	392.5 (111.1-1911.4)	203.53 (92.3-823.9)	<0.001
		203.53 (92.3-823.9)	
*Median investigation costs(range)*	147.4 (40.7-515.4)	93.9 (37.5-528.6)	0.03
*Median total medication costs (range)*	347.6 (87.6-1753.9)	372 (57.8-1698.9)	0.13
*Median antibiotics costs (range)*	82.5 (8.9-269)	39.1 (0.28-173.7)	0.004
*Median Carbapenem costs (range)*	35.73 (0–218.3)	12.9 (0–146.8)	0.0002
**After infection**			
*Median laboratory costs (range)*	412.4 (53.76-4029.4)	109.8 (57.3-1131.6)	<0.001
*Median investigation costs (range)*	109.2 (8.6-559)	64.1 (0–279.2)	0.002
*Median total medication costs (range)*	584.4 (91.4-9633.6)	200.7 (53.2-1761.8)	0.0002
*Median antibiotics costs (range)*	146.1 (15.7-3491.9)	39.5 (0.85-572.9)	0.0001
*Median Carbapenem costs (range)*	56.38 (0–2040.88)	10.47 (0–211.98)	<0.001

### Length of stay analysis

There were 35 survivors among nosocomial ICU RGNB acquired infected patients. A comparison of the length of stay in the hospital after ICU admission between the Cases and Controls A showed that cases infected with RGNB stayed longer in the hospital. The details are shown in Table 5.

**Table 5:** **Comparison of length of stay between the propensity matched cases and controls A**

**Survivors**	**RGNB (n = 35)**	**no GNB (n = 35)**	**p value**
**Total hospital stay**			
*Median days in hospital (range)*	41 (13–301)	22 (7–198)	0.01
**After admission to the ICU**			
*Median days in hospital (range)*	37 (13–301)	17 (2–107)	0.01

Among the 35 survivors, 21 of them had no SGNB isolated during the current admission and were included in the analysis to assess the difference in the LOS. When the cases were compared with patients infected with SGNB (Control B), we found that the there was no difference in the total length of stay at the hospital, after admission to the ICU and after Infection between the RGNB infected cases and SGNB infected Control B (Table 6).

**Table 6:** **Comparison of length of stay between the propensity matched cases and controls B**

**Survivors**	**RGNB (n = 21)**	**SGNB (n = 21)**	**p value**
**Total hospital stay**			
*Median days in hospital (range)*	34 (13–301)	43 (7–156)	0.39
**After admission to the ICU**			
*Median days in hospital (range)*	34 (13–301)	42 (7–124)	0.41
**After infection**			
*Median days in hospital (range)*	24 (2–294)	33 (1–97)	0.29

## Discussion

Our propensity matched case control study showed that patients with nosocomial ICU acquired RGNB infection encountered markedly increased hospital costs when compared with patients with no GNB infection and those with SGNB infection. The median per day costs of laboratory tests and antibiotics were 1.5-2 times higher than that of patients with no GNB infection and were nearly 3 times more than for SGNB infected patients.

In a previous analysis of ICU infections, nearly 80% of the hospital costs were contributed by an ICU stay of more than 5 days [19]. In our study, we found that the costs of RGNB infection were higher even after adjusting for the length of stay in the ICU. We also did not find an increase in length of stay for patients infected with RGNB compared with patients infected with SGNB. This suggests that it is the costs of treatment rather than simply the prolonging of hospitalization that increases the cost of antimicrobial resistance. While the economic impact of MRSA has long been recognized, the importance of resistant gram-negative bacilli is only recently being recognized. A retrospective study from Austria found that the total hospital costs for patients infected with RGNB were significantly higher than for patients with MRSA partly because of the costs of ICU care [20]. A retrospective study from Spain, also showed that the total hospital costs of patients with resistant and multidrug resistant *Pseudomonas aeruginosa* were 1.4 times and 1.7 times more than those patients with the non-resistant *Pseudomonas aeruginosa* [21]. In our study, we have noted a comparable rate of increased costs associated with nosocomial ICU acquired RGNB infection. While there are many new drugs recently licensed for the treatment of resistant gram-positive infections, no new agents are available for multi-resistant gram-negative infections. Older drugs such as colistin are increasingly being used as well as costly combinations of drugs.

Overall, in our propensity score matched cost analysis we found that nosocomial multi-resistant infections add significantly to the already heavy financial burden of patients in the ICU and their providers in Asia as has been previously reported in Europe and North America [22-24]. A recent study involving patients with RGNB bacteremia done in Singapore at two tertiary care hospitals showed that RGNB bacteremia contributed significantly to higher hospitalization costs for patients in general. The bulk (62.3%) of the excess cost was paid for by government subvention [25].

Although clinicians especially those working in ICUs have long recognized increasing antimicrobial resistance rates and decreasing options for treatment, this has not been widely recognized in the wider medical community and more importantly among funders and policy makers. The World Alliance Against Antibiotic Resistance (WAAAR), Inter Academy Panel and Inter Academy Medical Panel (IAP-IAMP), ReAct, Infectious Diseases Society of America(IDSA) and Alliance for the Prudent Use of Antibiotics (APUA) are some of the global alliances assembled by academics and clinicians to try to reduce antimicrobial resistance [26]. Some government linked agencies such as the Centers for Disease Control and Prevention(CDC), WHO and the European Commission [27-29] have also devised strategies to reduce antimicrobial resistance but the WHO has pointed out that the efforts are often fragmented and lack a solid evidence base [30]. Partnership with politicians and policy makers is crucial to mobilize resources to control the spread of drug resistance. Quality data demonstrating the economic impact of these resistant organisms would help to convince policy makers to invest in cost effective control measures to contain them. With rising healthcare costs in almost every country worldwide, these are very important considerations. Governments and funders need to be aware of these costs which could be the impetus for novel funding mechanisms to support the development of much needed therapeutics for RGNB. Economic analyses have helped drive public policy in other areas. For example, screening for colorectal cancer has been shown to be cost effective and also more importantly to reduce mortality [31]. This has now become national policy in most developed countries.

The increased costs attributable to the presence of nosocomial drug resistant gram negative bacilli infections among critically ill patients that we have documented helps make the case that there is a strong economic imperative to reduce these infections. With fewer antibiotics in the pipeline for drug resistant gram negative infections, controlling their incidence and spread becomes even more important [32].

To our knowledge, no one has previously examined the economic impact of RGNB infections in an ICU population using a propensity score approach. As it would be impossible and unethical to randomize patients to be infected with multi-resistant organisms, the main modality used to study these infections has been the case control approach [22,33]. This has been fraught with possible confounders and bias. In order to select a control group with similar characteristics to the RGNB infected patients, we employed a propensity score matching technique to reduce bias in the analysis. This has been shown in other settings to be useful [34]. We intended to capture only those patients with ICU acquired nosocomial resistant gram negative infection as this has been the trigger for calls for broad spectrum initial antibiotic therapy for all patients with the systemic inflammatory response syndrome in ICUs. The actual number of these patients with a proven resistant ICU acquired gram-negative infection turned out to be low despite reviewing nearly 2000 ICU admissions and hence we ended with a small sample size. Larger multi-center studies should be conducted to validate our findings.

Other potential limitations are that the study was done at a single center and we measured only the direct costs borne by the patient and funders. We did not include the indirect costs including the, opportunity costs such as loss of bed days for the hospital or the additional costs of follow-up outpatient visits and loss of earnings for the patient.

## Conclusion

Nosocomial acquired RGNB infections increase the total hospital costs in ICU patients. Urgent measures need to be taken to design cost-effective strategies to decrease the spread of these drug resistant infections.

## Abbreviations

ICU: Intensive Care Unit
MICU: Medical Intensive Care Unit
SICU: Surgical Intensive Care Unit
GNB: Gram Negative Bacilli
SGNB: Susceptible Gram Negative Bacilli
RGNB: Resistant Gram Negative Bacilli
WAAAR: World Alliance Against Antibiotic Resistance
IAP-IAMP: Inter Academy Panel and Inter Academy Medical Panel
IDSA: Infectious Diseases Society of America
APUA: Alliance for the Prudent Use of Antibiotics
WHO: World Health Organization
